# Assessment of the distribution of human and material resources for eye health in the public sector in Nampula, Mozambique

**DOI:** 10.1186/s12960-023-00812-w

**Published:** 2023-03-31

**Authors:** Dulnério Barbosa Sengo, Zubair Momade Abudo Salamo, Isaura Ilorena d’Alva Brito dos Santos, Laura Mavota Mate, Sancho Manuel Chivinde, Raul Moragues, Pablo Caballero Pérez, Inmaculada López-Izquierdo

**Affiliations:** 1 Faculdade Ciências de Saúde, Universidade Lúrio, Bairro de Marrere, R. nr. 4250, Km 2,3, Nampula, Mozambique; 2 Departamento de Enfermería Comunitaria, Medicina Preventiva y Salud Pública e Historia de la Ciencia, Universitat d’Alacant, Carretera Sant Vicent del Raspeig s/n, 03690, Sant Vicent del Raspeig, Alacant, Spain; 3 Ministério dos Combatentes, Av Mártires Machava 307, Cidade de Maputo, Moçambique; 4 Hospital Central de Nampula, Av Samora Machel, Bairro Central, Cidade de Nampula, Moçambique; 5 Departamento Estadística, Matemáticas e Informática, Universitas Miguel Hernandez, Av de la Universidad s/n, 03202 Elx, Spain; 6 Departamento de Física de la Materia Condensada, Universidad de Sevilla, Av. Reina Mercedes s/n, 41012 Sevilla, Spain

**Keywords:** Eye health professionals, Ophthalmologist, Optometrist, Allied ophthalmic personnel, Eye health services, Equipment, Distribution, Nampula, Mozambique

## Abstract

**Background:**

The unavailability of human and material resources can affect access to eye health services, constituting an obstacle in the fight against avoidable visual impairment. This study aimed to assess the availability and distribution of human and material resources for eye health in the public sector in Nampula province.

**Methods:**

A mixed method approach was used, which included document reviews (to extract information regarding the number of professionals and inhabitants in each district) and application of a questionnaire to heads of the ophthalmology department in each health facility (to obtain the list of available equipment). The ratios of eye health professionals per population in Nampula province and each of its districts were calculated and evaluated taking into account the recommendations of the World Health Organization (WHO). Based on the level of care of each health facility, the availability of equipment was evaluated.

**Results:**

Nampula Province has not reached the recommended ratio of eye health professionals per population in the different categories (ophthalmic technicians with 0.8 per 100 thousand inhabitants; optometrists and ophthalmologists with 0.4 and 0.2 per 250 thousand inhabitants, respectively). Most districts of Nampula did not reach the recommended ratio in the three categories of professionals, except Nampula City (provincial capital). However, there was a greater concentration of professionals and facilities with eye health services in the provincial capital. Primary and secondary level health facilities lacked some equipment to provide eye health services within their scope.

**Conclusions:**

There is an unequal distribution of the workforce in Nampula and the centralization of surgical services at the Central Hospital of Nampula level. Therefore, there is a need to review resource distribution strategies and decentralization policy of eye health services in Nampula.

**Supplementary Information:**

The online version contains supplementary material available at 10.1186/s12960-023-00812-w.

## Introduction

It is globally estimated that 2.2 billion people have visual impairment (VI) or blindness, among these, at least 1 billion could have been treated or prevented if they had received adequate care [1]. The high prevalence of VI often comes from the unavailability, limitation, and restricted access to eye health services [2]. Africa is a great example of this, as less than 1% of the global number of ophthalmologists work in Africa and only 13 African countries have achieved the recommended minimum number of eye care professionals per population, which is reflected in the high burden of VI on the continent (with an estimated 4 0.8 million blind and 16.6 million visually impaired) [3].

The density of health professionals in a given region is considered one of the main indicators of the availability and accessibility of health services in that region [1, 3, 4]. In general, the most remote and poor areas of low-income countries have less access to eye care due to a shortage of health professionals with adequate skills and unequal distribution of resources [3].

The Global Initiative for the Elimination of Avoidable Blindness “VISION 2020: the Right to Sight” defined as a priority the development of specialized human resources for eye care and the monitoring of the proportion of professionals per population, as one of the essential pillars for achieving universal eye health until 2020 [5, 6]. Universal coverage of eye health services is only possible with the right number of qualified professionals and adequate distribution of them, thus guaranteeing the quality of eye care, its treatment, and, therefore, prevention of blindness [7, 8].

The Global Action Plan (GAP) 2014–2019 focused on ensuring access to quality eye care for all (including the poor, minorities, and disabled) and a 25% reduction in the prevalence of preventable VI by 2019 [6]. Despite efforts towards this initiative, uncorrected refractive errors and cataracts remain the major causes of preventable VI, so the global goals for eye health by 2030 are focused on increasing effective coverage of cataract surgery (eCSC) and effective coverage of refractive errors (eREC)[9]. Therefore, to meet these goals, human resources with skills to perform refraction and cataract surgeries such as ophthalmologists, optometrists and ophthalmic technicians (OTs) have been a priority [6, 10].

An ophthalmologist is a specialist postgraduate doctor in ophthalmology, trained to diagnose, treat (medical and surgically) and prevent eye diseases, using specialized procedures and techniques [3]. The training of ophthalmologists in Mozambique is carried out by the ophthalmology department of the Central Hospital of Maputo (CHM), Beira (CHB), and Nampula (CHN), with a minimum duration of 3 years.

An optometrist is a primary eye care and visual system professional who provides comprehensive eye and vision care that includes refraction and dispensing, detection or diagnosis and treatment of eye diseases, and the rehabilitation of visual system conditions [3]. The training of optometrists is carried out by Lúrio University through a degree course, lasting 4.5 years. The training of optometrists in Mozambique aims to achieve eye care category four (which includes the use of pharmaceutical agents and other procedures to manage eye conditions/diseases) [11, 12].

Ophthalmic technicians (OT) have the main task of identifying and treating common eye problems (such as conjunctivitis, blepharitis, dry eyes, or minor trauma), managing refractive errors, and prescribing spectacles, mainly in rural areas. The OTs are trained by the Health Sciences Institutes of Maputo, Beira, and Nampula through a mid-level course, lasting 18 months. To enroll in this course, you must be a nurse or medicine agent for the National Health System. Their skills are similar to the so-called ophthalmic nurses in other countries [3, 13].

The World Health Organization (WHO) recommends a population-based eye care professional distribution ratio of 1:250 000 for ophthalmologists and optometrists, and 1:100 000 for OTs [3, 6, 14]. In addition to the appropriate ratio of eye care professionals; infrastructure, equipment, equipment maintenance, supplies, and technology are needed to provide quality care. Studies have shown that basic equipment and supplies have been lacking, thus compromising the quality of services provided [15].

The International Agency for the Prevention of Blindness (IAPB) has prepared lists of essential equipment for the provision of cataract surgery, refraction and low vision services, and management of glaucoma, diabetic retinopathy, and trachomatous trichiasis. In the absence of specific Mozambican guidelines, these lists are an alternative to assist in the planning, acquisition, and control of essential equipment for the provision of eye health services with the minimum recommended quality, taking into account the most common causes of VI, especially in regions with limited resources [16–21].

In Mozambique, to date, published studies with information regarding the distribution of human and material resources for eye health are scarce. In 2010, Mozambique had only 13 ophthalmologists and 34 OTs in the whole country (for a population of around 20 million inhabitants), so the ratio of professional per inhabitants was below the recommendations of the WHO [13, 22]. Since then, several initiatives have been implemented to reverse this scenario, with the introduction of the first training course for optometrists in Mozambique [23, 24], and the expansion of ophthalmologist training programs.

Therefore, this study aims to assess the current situation in Nampula province concerning the availability and distribution of human and material resources for eye health care in the public sector in Nampula, Mozambique. This information will be useful to design appropriate strategies and improve human and material resource management policies for eye health, to achieve minimum requirements for the provision of quality eye health services for everyone.

## Materials and methods

A mixed method approach was used, which included desk-based reviews and a questionnaire-based survey in health facilities with eye health services in Nampula province in 2021.

### Study location

Nampula province is the most populous province in Mozambique, with 20.6% of the population (5 483 382 inhabitants). It is located in the north of Mozambique, with an area of 81 606 km^2^ and a population density of 78.2 inhabitants per km^2^. Most of the population of Nampula lives in rural areas (67.8%). Nampula Province is divided into 23 districts, and Nampula City is its provincial capital [25, 26].

The National Health System in Mozambique is organized into 4 levels of service delivery (primary level: Health Posts and Health Centers; secondary level: Rural, District, and General Hospitals; tertiary level: Provincial Hospitals; and quaternary level: Central and Specialized Hospitals) [27, 28]. The primary (Health Posts and Health Centers) and secondary (Rural, District, and General Hospitals) levels are dedicated to Primary Health Care. The tertiary (Provincial Hospitals) and quaternary (Central Hospitals) levels offer secondary and tertiary health care [27]. Nampula Province contains 240 health facilities, of which 95 are Health Posts, 137 Health Centers, 3 District Hospitals, 3 Rural Hospitals, 1 General Hospital, and 1 Central Hospital.

Primary eye care services focus on the diagnosis and treatment of simple eye conditions, refraction services, emergency detection and referral, cataracts and other causes of visual impairment, and rehabilitation services. Secondary eye care services are dedicated to the diagnosis and management of the main causes of VI, in addition to refractive errors. These services include surgery (cataracts and glaucoma) and laser and injection therapies (for diabetic retinopathy and age-related macular degeneration). Tertiary eye care services comprise a range of sub-specialized services for the management of more complex eye conditions and training programs [29].

### Definition of variables

The human resources involved are the main categories of eye health professionals in Mozambique: ophthalmologists, optometrists, and OTs. The ratio of eye health professionals per inhabitant was estimated for each group (ophthalmologists, optometrists, and OTs) at provincial and district levels in Nampula province, taking into account the ratios recommended by the WHO (1:250 000 or 4:1 million inhabitants for ophthalmologists and optometrists, and 1:100 000 or 10:1 million inhabitants for OTs) [3, 6, 14].

The balance of human resources is the result of the relationship between the number of existing professionals and the ideal number of professionals. Thus, negative values express a deficit of professionals (the number of eye health professionals in short supply to reach the recommended ratio) and positive values express an excess of professionals (number of professionals above the recommended) by province and district.

Regarding equipment for eye health, a list was made taking into account the standard and essential equipment determined by the International Agency for the Prevention of Blindness (IAPB) [17–21]. This includes equipment for services of refraction, diagnosis and management of glaucoma, cataract, diabetic retinopathy, and trachomatous trichiasis. Only operational equipment was included in the study.

### Data collection and research tool

Data collection took place through document analysis and the application of a questionnaire to the heads of the ophthalmology department in each health facility. Information on the number of inhabitants at provincial and district levels was extracted from the Definitive Results of the 2017 General Population and Eligibility Census [26]. Information on the number of eye health professionals in the public sector in each district of Nampula province was obtained from the Annual Report of the Eye Health Program 2021 [30].

Through the Nampula Provincial Directorate of Health, all health facilities with eye health services across Nampula province and the respective heads of the ophthalmology department in each health facility were identified. A list of all facilities with eye health services, their heads, physical address, and contact (telephone and email) was prepared.

The questionnaire on human resources, infrastructure, and equipment available developed by WHO and IAPB [31] was adapted taking into account the standard list of essential equipment (for refraction services, management of glaucoma, cataract, diabetic retinopathy, and trachomatous trichiasis) of the IAPB [17–21]. A qualitative pre-test interview was previously conducted with four eye health professionals with at least five years of experience (one ophthalmologist, one OT and two optometrists) who were not heads of the ophthalmology department of any of the health facilities.

The questionnaire's final version was structured, self-administered and not validated (see Additional file 1). It was applied to the heads of the ophthalmology department of health facilities with eye health services to collect information on the material resources for eye care available in each health facility. The questionnaire and the respective informed consent form were shared by email with each interviewee. Data collection took place from April 2021 to November 2021.

### Ethical aspects

This study was previously authorized by the Provincial Health Service of Nampula and the Provincial Health Directorate of Nampula, and later by the Institutional Committee of Bioethics for Health of Lúrio University, with the ref: 05/Feb/CBISUL/21, on 25 February 2021. All interviewees were previously informed about the nature of the study and participated in the study by signing an informed consent form. Therefore, this study followed the principles of the Declaration of Helsinki.

### Statistical analysis

The types of equipment were grouped into 4 categories (refraction services, cataract surgery, glaucoma, diabetic retinopathy, and trachomatous trichiasis management) according to the IAPB Standard Equipment List [17–21, 32]. The calculation of the ratio of professionals resulted from dividing the number of inhabitants by the number of professionals in each category. To calculate the balance of human resources in every province and each district of Nampula, the subtraction operation was used, where the number of existing professionals was subtracted by the ideal number as recommended by the WHO [3, 6, 14].

A database was created using Microsoft Excel (version 2010). Data were extracted from each completed questionnaire and entered into the database by two researchers (D.B.S and Z.M.A.S). The formulas for calculating the ratio and balance of eye health professionals were entered into the database. Using simple descriptive statistics, the proportions of equipment available at different levels of health care were identified.

## Results

Twenty-six department heads of ophthalmology from the 26 health facilities with eye health services were asked to answer the questionnaire, and all agreed to participate in the study. Participants were aged between 32 and 58 years (with an average of 38.5 years of age), the predominant gender was male (76.9%), almost all were OTs (96.2%) and only one was an ophthalmologist (3.8%).

### Human resources for eye care

Overall, Nampula Province has not reached the recommended ratio of eye health professionals per population in the different categories. The current numbers should be increased by 9 OTs, 14 optometrists, and 18 ophthalmologists to reach the recommended ratios. However, there was also an uneven distribution of eye health professionals across the province, with a greater concentration of professionals in the provincial capital (Nampula City), so most districts did not reach the recommended ratio, presenting a negative balance. Nampula City is the district with the best ratio of professional per inhabitants (in all categories), with a positive balance (Table 1).

**Table 1:** Distribution of eye health professionals in Nampula Province

Districts	Population	Ophthalmic technicians			Optometrists			Ophthalmologists		
		Actual *N*°	Rate per 100 thousand population	Balance	Actual *N*°	Rate per 250 thousand population	Balance	Actual *N*°	Rate per 250 thousand population	Balance
Angoche	347 176	1	0.3	− 2	1	0.7	0	0	0	− 1
Erati	387 713	2	0.5	− 2	1	0.6	− 1	0	0	− 2
Island of Mozambique	64 577	1	1.5	0	0	0.0	0	0	0	0
Lalaua	98 177	0	0.0	− 1	0	0.0	0	0	0	0
Larde	174 641	0	0.0	− 2	0	0.0	− 1	0	0	− 1
Liupo	107 369	1	0.9	0	0	0.0	0	0	0	0
Malema	98 385	1	1.0	0	0	0.0	0	0	0	0
Meconta	89 259	1	1.1	0	0	0.0	0	0	0	0
Mecuburi	213 011	1	0.5	− 1	0	0.0	− 1	0	0	− 1
Memba	223 760	1	0.4	− 1	0	0.0	− 1	0	0	− 1
Mogincual	207 285	0	0.0	− 2	0	0.0	− 1	0	0	− 1
Mogovolas	328 460	1	0.3	− 2	0	0.0	− 1	0	0	− 1
Moma	368 905	1	0.3	− 3	0	0.0	− 1	0	0	− 1
Monapo	324 442	2	0.6	− 1	0	0.0	− 1	0	0	− 1
Mossuril	393 813	0	0.0	− 4	0	0.0	− 2	0	0	− 2
Muecate	134 280	0	0.0	− 1	0	0.0	− 1	0	0	− 1
Murrupula	184 732	2	1.1	0	0	0.0	− 1	0	0	− 1
Nacala	287 536	2	0.7	− 1	1	0.9	0	0	0	− 1
Nacala-a-Velha	121 726	1	0.8	0	0	0.0	0	0	0	0
Nacaroa	145 450	1	0.7	0	0	0.0	− 1	0	0	− 1
Nampula City	760 214	25	3.3	17	4	1.3	1	4	1.3	1
Rapale	166 327	1	0.6	− 1	0	0.0	− 1	0	0	− 1
Ribaue	256 144	1	0.4	− 2	1	1.0	0	0	0	− 1
Total	5 483 382	46	0.8	− 9	8	0.4	− 14	4	0.2	− 18

### Health facilities with eye health services

**Fig. 1:**
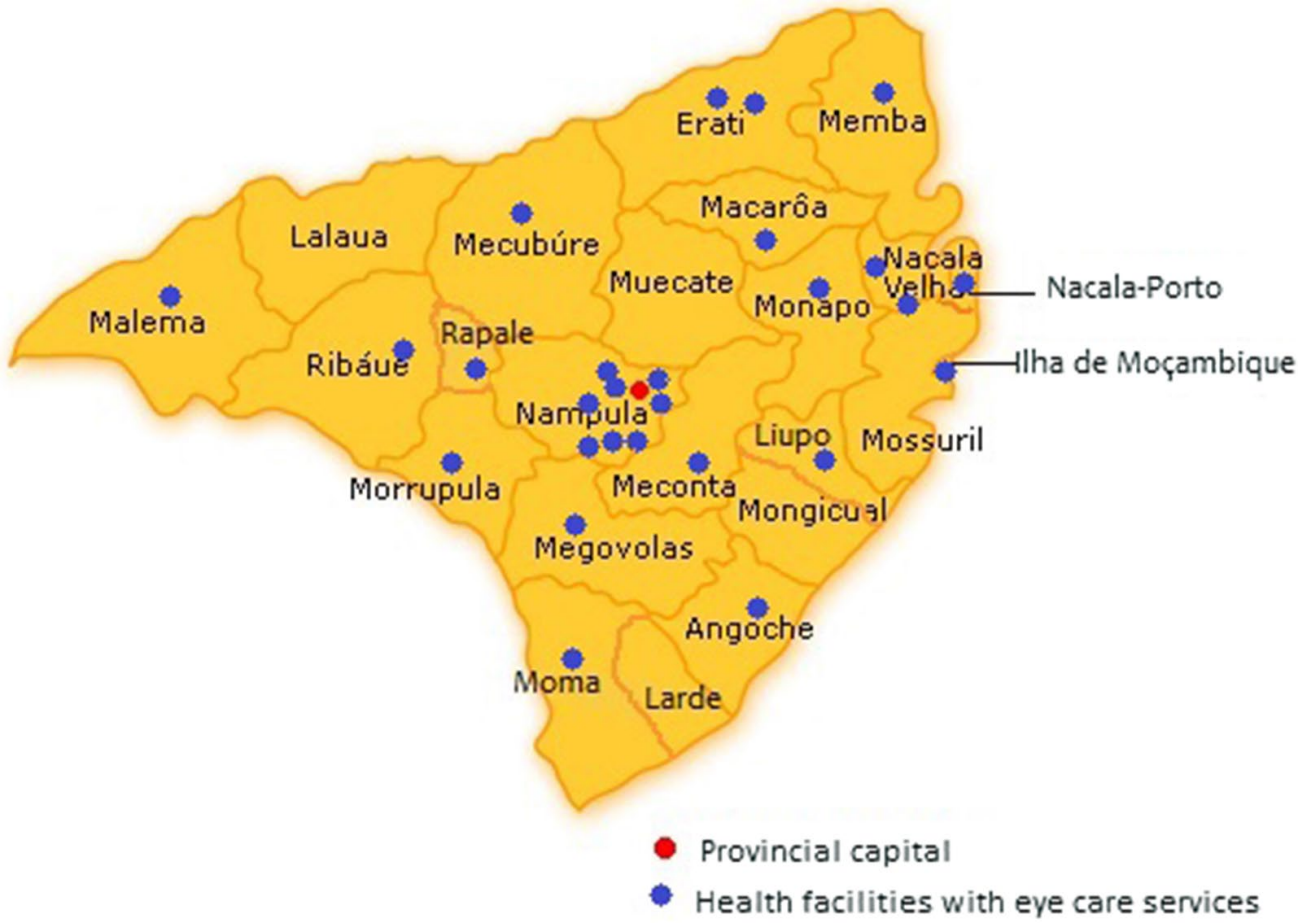
Distribution of public health facilities with eye care services in Nampula province

Nampula province contains a total of 240 public health care facilities and only 26 (10.8%) provide eye health services. Most health facilities with eye health services are concentrated in Nampula City (with 8 public health care facilities with eye health services, corresponding to 30.8%), so the provincial capital has the best ratio of public health facility per inhabitants (Table 2 and Fig. 1). The districts of Moma, Angoche, Mogovolas, and Monapo have the worst ratio, and on the other hand, the districts of Lalaua, Larde, Mogincual, Mossuril, and Muecate do not have any health facilities with eye health services (Table 2), and therefore no eye health professionals (Table 1). Of a total of 26 public health care facilities with eye health services, 16 are primary level (Health Centers), 9 secondary level hospitals (2 Rural Hospitals, 6 District Hospitals, and 1 General Hospital), and one quaternary level hospital (Hospital Central de Nampula).

**Table 2:** Distribution of Health Care Facilities by District in Nampula Province

Districts	Public health care facilities	Public healthcare facilities with eye health services	Ratio of facilities with eye health services per inhabitants
Angoche	20	1	1: 347 176
Erati	11	2	1: 193 857
Island of Mozambique	5	1	1: 64 577
Lalaua	7	0	N/A
Larde	9	0	N/A
Liupo	3	1	1: 107 369
Malema	10	1	1: 98 385
Meconta	8	1	1: 89 259
Mecuburi	13	1	1: 213 011
Memba	14	1	1: 223 760
Mogincual	6	0	N/A
Mogovolas	8	1	1: 328 460
Moma	11	1	1: 368 905
Monapo	17	1	1: 324 442
Mossuril	12	0	N/A
Muecate	11	0	N/A
Murrupula	6	1	1: 184 732
Nacala	13	2	1: 143 768
Nacala-a-Velha	6	1	1: 121 726
Nacaroa	7	1	1: 145 450
Nampula City	25	8	1: 95 027
Rapale	8	1	1: 166 327
Ribaue	10	1	1: 256 144
Total	240	26	1: 210 899

### Material resources for eye care

We report the distribution of eye care equipment in Table 3. All health facilities contain visual acuity measurement charts, trial lens sets, and trial frames. However, there is a lack of certain equipment for providing refraction services such as retinoscopes, autorefractors, and lensometers in primary and secondary health facilities. Primary health facilities do not contain this equipment, and only 33.3% of secondary facilities have an autorefractor and streak retinoscope.

**Table 3:** Distribution of eye care equipment available in public health facilities

Equipment	Public healthcare facilities					
	Primary level		Secondary level		Quaternary level	
	(HC)		(RH/DH /GH)		(CHN)	
	*N* = 16		*N* = 9		*N* = 1	
	*N*	%	*N*	%	*N*	%
Autorefractor	0	0.0	3	33.3	1	100.0
Lensometer	0	0.0	0	0.0	1	100.0
Streak retinoscope	0	0.0	3	33.3	1	100.0
Visual acuity test (near)	16	100.0	9	100.0	1	100.0
Visual acuity test (distance)	16	100.0	9	100.0	1	100.0
Trial frame	16	100.0	9	100.0	1	100.0
Trial lens set	16	100.0	9	100.0	1	100.0
Slit lamp biomicroscope	2	12.5	4	44.4	1	100.0
Fundus lens	0	0.0	0	0.0	1	100.0
Indirect ophthalmoscope	0	0.0	0	0.0	1	100.0
Direct ophthalmoscope	8	50.0	7	77.8	1	100.0
Ultrasonography (A or A/B scan)	0	0.0	0	0.0	1	100.0
Keratometer	0	0.0	3	33.3	1	100.0
Schiotz tonometer	9	56.3	6	66.7	1	100.0
Applanation tonometer	0	0.0	0	0.0	1	100.0
Gonio lens	0	0.0	0	0.0	1	100.0
Operating microscope	0	0.0	0	0.0	1	100.0
Vitrectomy Machine	0	0.0	0	0.0	1	100.0
Cataract surgical set	0	0.0	0	0.0	1	100.0
Binomag Loupe Binocular	0	0.0	0	0.0	1	100.0
Visual field analyser	0	0.0	0	0.0	1	100.0
Optical coherence tomography	0	0.0	0	0.0	1	100.0
YAG laser	0	0.0	0	0.0	1	100.0
Trabeculectomy set	0	0.0	0	0.0	1	100.0
Non-mydriatic fundus retinography	0	0.0	0	0.0	1	100.0
Pen torch	14	87.5	7	77.8	1	100.0
Hand magnifying lens	14	87.5	7	77.8	1	100.0
Epilation forceps	11	68.8	7	77.8	1	100.0
Surgical set for TT	0	0.0	0	0.0	1	100.0

*HC* health center, *RH* rural hospital, *DH* district hospital, *GH* general hospital, *CHN* Central Hospital of Nampula

The facilities at the primary level are quite limited in terms of diagnostic equipment for eye pathologies such as cataracts, glaucoma, and diabetic retinopathy (only 12.5% have a slit lamp, 50% a direct ophthalmoscope and 56.3% a Schiotz tonometer). At the secondary level, most facilities have minimal equipment for the diagnosis of ocular pathologies (44.4% have a slit lamp, 77.8% have a direct ophthalmoscope and 66.7% have a Schiotz tonometer). The Central Hospital of Nampula (CHN) has the best conditions not only for the diagnosis, but also for the surgical treatment of these conditions.

Regarding trachomatous trichiasis, most facilities are equipped for diagnosis, as most have a pen torch, hand magnifying lens or even a slit lamp. However, only the CHN is properly equipped for surgical treatment, while the primary and secondary facilities have equipment only for non-surgical interventions (most contain epilation forceps).

## Discussion

The WHO's GAP 2014–2019 focused on achieving universal coverage of eye health services and reducing blindness and VI by 25% [33]. However, it is impossible to speak about universal coverage without mentioning the availability of human and material resources throughout the different regions of the country. Therefore, in order to assess and monitor progress, it is important to control the density of health workers in a given region, taking into account the population size (ratio of eye care professionals per inhabitants) and the availability of essential materials for the provision of eye health services in that region.

Currently, the critical shortage of human resources is recognized worldwide as one of the main barriers to achieving progress in health and the goals of sustainable development. In particular, Mozambique is on the list of countries identified with a critical shortage of human resources for health [7].

### Human resources for eye care

Overall, Nampula Province has not achieved the recommended ratio of eye care professionals to population in any of the classes (OTs, optometrists and Ophthalmologists). Nampula City (capital) was the only district with a recommended ratio in the three classes of professionals and a positive balance, resulting from the greater concentration of eye health professionals in the capital. This shows a great disparity between the capital and regions outside the capital (other districts, which, mostly, had a negative balance).

In the study carried out in the province of KwaZulu-Natal, South Africa [6], optometrists were the only class that reached the recommended ratio, while ophthalmologists and ophthalmic nurses presented a ratio far from the WHO recommendations. This difference may be due to the fact that South Africa has more optometrist training institutions and has been training optometrists longer than Mozambique, and KwaZulu-Natal had its first optometrist training institution in 1979 [34].

On the other hand, in the study carried out in Ogun State, Nigeria [35], ophthalmologists were the only class with the recommended ratio (5.4 per million inhabitants), while optometrists and ophthalmic nurses had a ratio below the recommended (with 3.8 and 6.3 per million inhabitants, respectively). This difference may be because Ogun State has 3 training programs for ophthalmologists and none for optometrists and ophthalmic nurses.

However, in the three studies there was a greater concentration of eye health professionals in the capital (urban area) [6, 35], which denotes an unequal distribution throughout the territory. We must not forget that in the three provinces (Nampula, KwaZulu-Natal and Ogun State) the population mostly lives in rural areas (3.715.849 people in Nampula live in rural areas, corresponding to 67.8% of its population) [26], so a large part of its population is left disadvantaged in eye health care, which can exacerbate the burden of eye diseases and VI in communities.

### Health facilities with eye health services

The province of Nampula has a total of 240 public health facilities. Out of these, only 26 (10.8%) provide eye health services, a relatively low percentage that may indicate poor investment in eye health in the public sector. Obviously, the sector is reinforced by the private sector, however the use of private eye health services entails costs and a large part of the population of Nampula does not have economic conditions to access them, as 55% of the population of Nampula lives in severe poverty (with less than 1.9 dollars a day) [36]. Therefore, the demand for eye health services in the public sector is greater. This results in overcrowding in public hospitals, longer waiting time in health facilities or in long distances to travel to enjoy eye health services, with additional transport costs. These factors constitute barriers to access to eye health services and a major obstacle in the fight against preventable and treatable VI [37].

### Material resources for eye care

The primary level facilities (Health Centers) have sufficient equipment for subjective assessment of refractive error, and most have the minimum conditions for preliminary screening of ocular pathologies. Trachomatous trichiasis can be managed non-surgically by 68.8% of health centers (as they contain epilation forceps). In studies carried out in Nigeria, health centers and health posts had only visual acuity tests and flashlights [38, 39]. Therefore, the health centers in Nampula were better equipped compared to the health centers in Nigeria. The equipment level of the facilities depends a lot on the guidelines and internal legislation of each country. However, taking into account that refractive errors are the main cause of VI in the world [40], health centers, being within reach of most communities, can be strategic to ensure greater coverage of refractive services. Considering that refractive services are part of the scope of primary eye care according to the framework for delivering eye health services integrated into the health system recommended by the WHO [29, 41, 42], it would be opportune for refractive interventions to be more effective at the community level that these facilities should be better equipped to provide refractive services.

The facilities at the secondary level (Rural, District, General Hospitals) are better equipped than those at the primary level (Health Centers), as was already supposed. However, a lack of important equipment was noted, such as the autorefractor and the Streak retinoscope, which are present in only 40% of the facilities. Most facilities have equipment that can detect eye conditions such as cataracts, glaucoma, diabetic retinopathy and trachomatous trichiasis. However, they do not have the capacity for (surgical) treatment and follow-up, and these patients must be referred to the CHN.

Similar results were found in Ghana [32], where most district, municipal and metropolitan hospitals did not have autorefractors and Streak retinoscopes. Some facilities were equipped to provide cataract surgery, although with certain limitations.

In Mozambican health legislation, secondary level facilities are also dedicated to primary health care, and at this level, it was expected that refraction services would be more complete. However, the lack of an autorefractor and a retinoscope makes it impossible for these facilities to perform objective refraction, limiting them to subjective assessment, which negatively impacts the effectiveness and quality of refraction services.

The CHN is the only hospital that provides tertiary eye care in the entire northern region of Mozambique (which involves Niassa, Cabo Delgado, and Nampula). It is very well equipped for refraction services and the diagnosis, treatment and follow-up of the most diverse eye pathologies. It is the only facility capable of performing surgery in cases of cataracts, glaucoma, diabetic retinopathy, and trachomatous trichiasis in Nampula, which shows the centralization of eye surgery services.

### Final considerations and recommendations

The centralization of services is the result of insufficient human and material resources, as well as their poor distribution. This results in increasing differences and inequalities between groups, thus violating one of the basic principles of the right to health, which is equity in health. The Mozambican Constitution (2004) enshrines the right to medical care for all citizens. It also guarantees that all citizens enjoy the same rights, regardless of color, race, ethnic origin, place of birth, religion, level of education, social position, or gender [43]. Therefore, it is the Government's responsibility to ensure accessibility to eye health services, especially for vulnerable groups and in remote areas.

As a way to increase the coverage of services, a team of health professionals from the CHN has periodically visited more remote regions in order to provide outpatient care and carry out surgery campaigns (especially for cataracts and trachomatous trichiasis). However, around 4.723.168 inhabitants living outside the capital (86% of the population of Nampula) can stay without access to these services for long periods, and this method may discourage the development of local eye care services [29].

Therefore, it is necessary to train the local workforce, as well as to equip the respective facilities with essential equipment to provide the most diverse services, especially surgical ones. As an example, some studies have shown encouraging results about cataract surgery performed by non-physician cataract surgeons after training. It has been a measure adopted mainly in low- and middle-income countries to compensate for the lack of ophthalmologists in remote areas [44].

Telemedicine and artificial intelligence can also be seen as an alternative. Despite the requirements for its operation, these have the potential to alleviate the lack of specialized health professionals and make services available in remote regions. Therefore, they can be an option in the medium and long term [45, 46].

Therefore, the solution does not only involve training more human resources. Future studies are needed that seek to analyze strategies and policies for the allocation of resources for eye care and retention of staff. These are factors that dictate the preference or choices of professionals with respect to their allocation or migration to other workplaces (rural to urban, and vice versa), as well as labor market trends.

However, for the effectiveness and quality of eye health services, more elements, in addition to human and material resources, need to be considered. These include financial resources, the information system, availability of supplements, and aspects related to leadership and service of delivery. Therefore, future studies that evaluate the other elements are equally necessary, since the proper functioning of the health system depends on the conjuncture of all these elements.

## Limitations

In this study, the availability of material resources for eye health in public health facilities was assessed using a non-validated self-administered questionnaire. Therefore, the researchers did not travel to the facilities to check the equipment closely, which can lead to some inaccuracy in the data, as there is a risk of self-report bias [47].

This article was focused only on the public health sector. It must be considered that private facilities have been supplying a part of the demand for eye health services, the ratios, especially in urban areas, can be higher and the differences between rural and urban areas can be even bigger.

## Conclusion

The quantity and distribution of eye health professionals, as well as the availability of material resources, are important health indicators in a given region, as they will directly reflect on the coverage and quality of services. There is indeed a lack of human resources for eye health in Nampula, which is aggravated by its poor distribution. While the capital (Nampula City) has a positive balance of professionals, that is, a number above the recommended, some provinces do not even have a professional. There are also limitations in terms of equipment in primary and secondary health facilities, due to the centralizing most services, especially surgical services, at the CHN, compromising coverage and access to these services by the community. Therefore, strategies for the distribution of human and material resources in eye health need to be rethought by health managers to have a more equitable distribution and guarantee access to eye health care for all and at all levels.

## Supplementary Information

**Additional file 1.** Questionnaire on the availability of material resources for eye health care

## Data Availability

The datasets used and analyzed during the current study are available from the corresponding author upon request.

## Abbreviations

VI: Visual impairment
GAP: Global Action Plan
OT: Ophthalmic technician
CHM: Central Hospital of Maputo
CHB: Central Hospital of Beira
CHN: Central Hospital of Nampula
WHO: World Health Organization
IAPB: International Agency for the Prevention of Blindness
HC: Health Center
RH: Rural Hospital
DH: District Hospital
GH: General Hospital
RF: Refraction
CT: Cataract
GC: Glaucoma
DR: Diabetic retinopathy
TT: Trachomatous trichiasis
