# Supplementary material for: Assessment of the distribution of human and material resources for eye health in the public sector in Nampula, Mozambique
Source: Hum Resour Health. 2023 Mar 31;21:27. doi: 10.1186/s12960-023-00812-w (PMC10067286; doi:10.1186/s12960-023-00812-w)
Supplement: Supplementary file 1 — Additional file 1. Questionnaire on the availability of material resources for eye health care [file 12960_2023_812_MOESM1_ESM.docx]

**Questionnaire on the availability of material resources for eye health care**

1. Personal information

1. Gender ___________________________
2. Birth date____________________________
3. Profession___________________________

2. Health Care Facility information

1. Health Care Facility name: _______________________________________________________
2. Type of Health Care Facility: _____________________________________________________
3. Location of the Health Care Facility: _______________________________________________

3. Below is the essential equipment list to carry out eye health care. Mark with an “X” the existing and operational equipment in your Health Facility.

| Equipment | Mark with "X" to confirm the existence |
| --- | --- |
| 1. Autorefractor |  |
| 1. Lensometer |  |
| 1. Streak retinoscope |  |
| 1. Visual acuity test (near) |  |
| 1. Visual acuity test (distance) |  |
| 1. Trial frame |  |
| 1. Trial lens set |  |
| 1. Slit lamp biomicroscope |  |
| 1. Fundus lens |  |
| 1. Indirect ophthalmoscope |  |
| 1. Direct ophthalmoscope |  |
| 1. Ultrasonography (A or A/B scan) |  |
| 1. Keratometer |  |
| 1. Schiotz tonometer |  |
| 1. Applanation tonometer |  |
| 1. Gonio lens |  |
| 1. Operating microscope |  |
| 1. Vitrectomy Machine |  |
| 1. Cataract surgical set |  |
| 1. Binomag Loupe Binocular |  |
| 1. Visual field analyser |  |
| 1. Optical Coherence Tomography |  |
| 1. YAG laser |  |
| 1. ARGON laser |  |
| 1. Trabeculectomy Set |  |
| 1. Non-Mydriatic Fundus Retinography |  |
| 1. Pen torch |  |
| 1. Hand magnifying lens |  |
| 1. Epilation forceps |  |
| 1. Surgical set for trachomatous trichiasis |  |
